# Improved quality of life in patients with no-option critical limb ischemia undergoing gene therapy with DVC1-0101

**DOI:** 10.1038/srep30035

**Published:** 2016-07-15

**Authors:** Takuya Matsumoto, Michiko Tanaka, Keiji Yoshiya, Ryosuke Yoshiga, Yutaka Matsubara, Kumi Horiuchi-Yoshida, Yoshikazu Yonemitsu, Yoshihiko Maehara

**Affiliations:** 1 Department of Surgery and Science, Graduate School of Medical Science, Kyushu University, 3-1-1 Maidashi, Higashi-ku, Fukuoka 812-8582, Japan; 2 R&D Laboratory for Innovative Biotherapeutics Science, Graduate School of Pharmaceutical Sciences, Kyushu University, Rm. 601, Collaborative Research Station I, 3-1-1 Maidashi, Higashi-ku, Fukuoka 812-8582, Japan

## Abstract

Critical limb ischemia (CLI) has a poor prognosis and adversely affects patients’ quality of life (QOL). Therapeutic angiogenesis may improve mobility, mortality, and QOL in CLI patients. However, the effectiveness of gene therapy on such patients’ QOL is unknown. DVC1-0101, a non-transmissible recombinant Sendai virus vector expressing human fibroblast growth factor-2 gene, demonstrated safety and efficacy in a phase I/II study of CLI patients. We investigated the effects of DVC1-0101 on QOL in this cohort. QOL was assessed using the Short Form-36 health survey version 2 (SF-36) in 12 patients at pre-administration, 28 days, and 3, 6, and 12 months post-treatment. We examined differences between pre and post-administration QOL scores and correlations between QOL scores and vascular parameters. Patients demonstrated low baselines scores on every SF-36 dimension. Post-treatment scores showed significant improvements in physical functioning at 3 and 6 months (P < 0.05), role-physical at 3, 6, and 12 months (P < 0.05), bodily pain at 1, 3, 6, and 12 months (P < 0.05), vitality at 1, 6, and 12 months (P < 0.05), and physical component summary at 6 and 12 months (P < 0.05). DVC1-0101-based gene therapy may improve QOL in CLI patients over a 6-month period.

Peripheral arterial disease (PAD) affects the arteries in the limbs and comprises a range of conditions caused by stenosis or occlusive atherosclerosis in the vascular bed^1^. PAD affects around 12% of the adult population and thus represents an important public health issue^2^. PAD progression is associated with reduced quality of life (QOL) due to limited mobility because of pain in the lower legs during walking (intermittent claudication). Intermittent claudication progresses to critical limb ischemia (CLI) in approximately one in four patients within 5 years^3^. CLI induces pain at rest and ischemic leg ulcers/gangrene, with substantial deterioration in QOL. Mortality is as high as 20%, and major amputation is required within 1 year in 40% of patients with CLI^4^. Although advanced surgical and endovascular techniques exist, many patients are ineligible^5^, and there is currently no effective pharmacologic therapy for CLI, resulting in the need for amputation in many patients.

The poor prognosis and increasing morbidity and mortality of CLI indicate the need for new therapies, including gene therapy, aimed at inducing angiogenesis^6^. The use of gene therapy to induce angiogenesis was initially proposed in the late 1990 s, and has since become a potentially attractive clinical option for the treatment of PAD^7^,^8^. Fibroblast growth factor-2 (FGF-2) is one of the most promising angiogenic growth factors. It was recognized as an angiogenic factor based on its induction of endothelial cell proliferation, migration, and morphogenesis, extracellular matrix degradation, and vessel maturation^9^. Furthermore, recombinant Sendai virus has been identified as an efficient gene carrier for FGF-2, allowing pronounced angiogenesis^10^,^11^,^12^. Unlike other gene-transfer virus vectors, Sendai virus is a cytoplasmic, negative-stranded RNA virus that replicates entirely in the cytoplasm of cells, with no DNA intermediate. DVC1-0101 is a new RNA gene drug based on a non-transmissible recombinant Sendai virus carrying the FGF-2 gene, which was investigated in a first-in-man gene therapy clinical trial in CLI patients who were ineligible for standard vascular interventions (‘no-option’ CLI) completed in March 2011^13^.

CLI patients experience significant limitations in terms of their physical functioning and functional status, thus, improving their QOL, as well as their clinical status, has become a vital treatment goal in these patients^14^. However, innovative gene therapy is still in the early clinical trial phase, and QOL data are therefore lacking. The current study investigated the effects of gene therapy using DVC1-0101 on QOL measures in 12 patients with CLI, initially treated in the above study^13^.

## Results

### Patient characteristics

A total of 12 patients were enrolled with a mean age of 65.0 ± 11.38 years (range 48–82 years), as described previously^13^. The majority of patients were male (83%) and 75% had a history of smoking. Ten patients were diagnosed with arteriosclerosis obliterans and two with thromboangiitis obliterans. Three patients had ulcers on their ankles and/or toes at enrolment. According to Fontaine grade, 75% were grade III and the rest were grade IV. SF-36 data were available for all 12 participants.

Baseline SF-36 data for all the participants are summarized in Supplementary Table S1. The mean (±standard deviation) pain level was 2.67 (±0.89) according to the 5-point Likert scale. The mean ankle–brachial index (ABI) was 0.49 (±0.31) mmHg, and the mean absolute claudication distance (ACD) based on the eight patients eligible for the treadmill test was 126.13 (±93.88) m. Of the 12 participants, nine had a medical history of re-vascularization, five had a history and/or comorbid myocardial infarction or angina, and six had a medical history of stroke.

### Norm-based QOL scores

**Figure 1:**
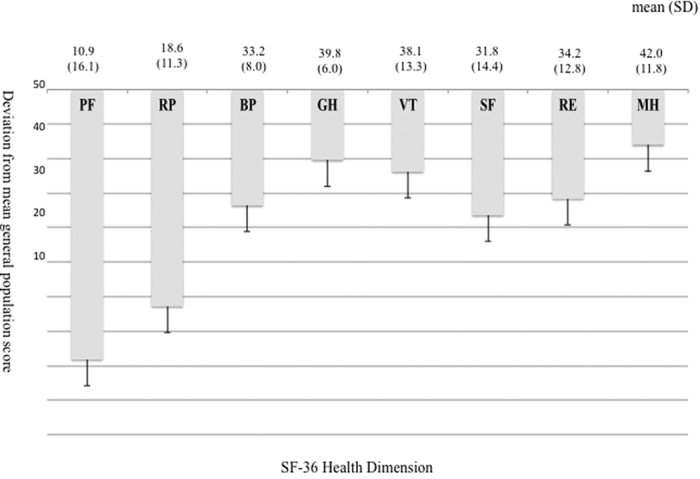
Differences in Short Form-36 health survey scores in 12 patients with critical limb ischemia compared with Japanese national standards. The Short Form-36 health survey includes eight domains: physical functioning (PF); role-physical (RP); bodily pain (BP); general health (GH); vitality (VT); social functioning (SF); role-emotional (RE); and mental health (MH). Abbreviation: SD, standard deviation.

All the patients demonstrated low scores in every dimension of SF-36 at baseline (Fig. 1). Mean norm-based scoring (NBS) scores were below those of the general population in all dimensions. The poorest scores obtained were in the physical health domain, including physical functioning and role-physical. The low physical component summary and role/social component summary scores reflected the patients’ overall poor physical health.

SF-36 NBS physical functioning and role/social component summary scores were significantly higher in TAO patients compared with ASO patients (P = 0.0302 and P = 0.0317, respectively). SF-36 NBS scores were also significantly lower in Fontaine grade III compared with grade IV in terms of vitality (P = 0.0122), role-emotional (P = 0.0295), mental health (P = 0.0441), and mental component summary (P = 0.0335) (Table 1).

### Effect of gene therapy on QOL score

Amputation strongly affects patient QOL, and the two patients who underwent amputation after administration of DVC1-0101 were therefore excluded from the analysis of treatment effectiveness (Table 2). There were significant improvements in physical functioning at 3 months (P = 0.0098) and 6 months (P = 0.042), in role-physical at 3 months (P = 0.0078), 6 months (P = 0.002), and 12 months (P = 0.0195), in bodily pain at 1 month (P = 0.0469), 3 months (P = 0.0039), 6 months (P = 0.0078), and 12 months (P = 0.0391), in vitality at 1 month (P = 0.0273), 6 months (P = 0.043), and 12 months (P = 0.0313), and in physical component summary at 6 months (P = 0.0322) and 12 months (P = 0.0068).

**Figure 2:**
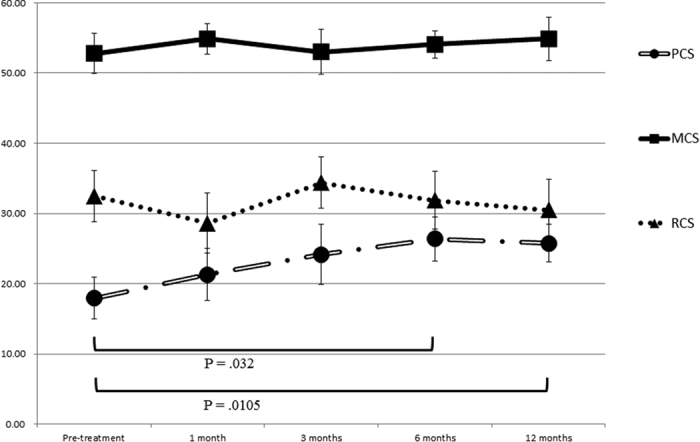
Overall summary measures of the Short Form-36 health survey for all 12 patients with critical limb ischemia. The eight domains of the Short Form-36 health survey can be aggregated into three summary measures: physical component summary (PCS); mental component summary (MCS); and role/social component summary (RCS).

Overall summary measures for all 12 participants revealed significant improvements in physical component summary at 6 months (P = 0.032) and 12 months (P = 0.0105) after administration of DVC1-0101 (Fig. 2). There was no dose-related effect of DVC1-0101 on QOL, except in terms of physical functioning and mental health, which both improved more in the low-dose (5 × 10^7^ and 2 × 10^8^ cell infections units/60 kg body weight) compared with the high-dose (1 × 10^9^ and 5 × 10^9^ cell infectious units/60 kg body weight) groups (Table 3).

### Correlations between QOL score and vascular parameters

We analysed the correlations between baseline factors and improvements in SF-36 measures after administration of gene therapy by paired *t*-tests. Age over 65 years was significantly associated with improvements in mental component summary (Supplementary Table S2). There were no differences in summary scores in relation to diagnosis, sex, Fontaine grade, medical history of myocardial infarction, or dose. There were no positive correlations between improvements in changes of SF-36 summary scores and changes of ACD, ABI, and pain scales after DVC1-0101 administration. There were, however, positive correlations between physical component summary and toe–brachial index (TBI) and thermography (r = 0.88 and r = 0.86, respectively; P = 0.001) (Supplementary Table S3).

## Discussion

Therapeutic angiogenesis for PAD using proteins, cells with angiogenic properties and angiogenic genes, has been examined extensively over the past two decades. However, no modality has shown definitive clinical benefit, including in terms of QOL. DVC1-0101 is a recombinant Sendai virus-based RNA gene drug that provides a novel gene-delivery system for strong expression of the native FGF-2 gene. Preclinical studies of DVC1-0101 have demonstrated local peak FGF-2 protein concentrations in muscles of >10-fold control levels^11^,^12^. FGF-2 was the first angiogenic factor shown to improve walking performance in PAD patients^15^ and Sendai virus-expressed FGF-2 demonstrated excellent limb-salvaging effects compared with other factors and vectors^11^,^12^. We therefore conducted a first-in-man phase I/IIa study to evaluate the safety and efficacy of DVC1-0101^13^. The current study assessed SF-36-based QOL in these patients and found that post-treatment QOL scores for physical functioning, role-physical, bodily pain, vitality, and physical component summary were significantly improved after administration of DVC1-0101. It must be noted that these results are extracted from a phase I/IIa, single-arm study, with no randomization and no-placebo control. Thus, the findings are preliminary and must be interpreted with caution; however, the results are suggestive of DVC1-0101 possibly showing some effectiveness at improving QOL in patients with no-option CLI.

PAD is associated with reduced walking ability, reduced physical and mental functional statuses, and QOL compared with patients without PAD^16^. Ischemic leg pain limits activities, and thus tends to isolate patients from social connections, with consequent stagnating effects on emotional and mental health. Baseline reference QOL data for patients with no-option CLI using SF-36 NBS reported low scores in every dimension^14^. Similarly, patients in the current study with no-option CLI demonstrated scores well below the mean for the general population in all dimensions. These low QOL scores suggest that patients with CLI need help to improve or preserve their QOL.

The severity of PAD has previously been reported to have a significant impact on QOL^17^. QOL is often indicated by walking performance in patients with intermittent claudication. A phase II, randomized, double-blind, placebo-controlled RAVE study concluded that there was no significant improvement in QOL in patients with predominantly unilateral intermittent claudication who underwent adenoviral vascular endothelial growth factor gene transfer compared with placebo, according to the walking impairment questionnaire and SF-36^18^. The phase II TRAFFIC study demonstrated that the primary outcome of change in peak walking time significantly increased in patients treated with single-dose compared with placebo, though improved walking ability did not affect QOL^15^. However, angiogenesis gene therapy is still in an experimental stage and results are based on early-phase clinical trials; further late-phase trials are therefore needed to examine the effects of angiogenesis on QOL in relation to walking function in patients with intermittent claudication.

Improving QOL for patients with CLI may be more challenging. QOL assessment using SF-36 in patients with CLI who underwent revascularization, major amputation, or conservative treatment failed to show any significant improvement^19^. Furthermore, although angioplasty significantly increased QOL scores in patients with intermittent claudication, no such improvement was seen in patients with CLI^20^. In contrast, Shigematsu *et al*.^21^ reported significant improvements in SF-36 measures, especially in terms of body pain and mental health dimensions, in CLI patients treated with hepatocyte growth factor plasmid compared with placebo in a phase III trial, with primary endpoints of rest pain and reduction of ulcer size^21^. The significant reductions in rest pain and ulcer size in this study suggest that QOL in terms of body pain and mental health dimensions increased.

Physical functioning scores improved significantly in the current study at 3 and 6 months after DVC1-0101 administration, and the physical component summary score improved significantly at 12 months. These findings suggest that it is possible that DVC1-0101 may effectively preserve or improve long-term QOL in patients with CLI. The factors associated with treatment-related improvements in QOL remain unclear. DVC1-0101 was shown to be safe, well-tolerated, and to improve walking function significantly^13^; however, the current study found no significant correlation between QOL and change in ACD. Furthermore, although an association between disease-related pain and QOL has been reported^20^, no such relationship was identified in the current study. Similarly, there was no dose-related effect of DVC1-0101 on QOL, suggesting that even low-dose DVC1-0101 may effectively improve or preserve long-term QOL in patients with CLI. The significant improvements observed in QOL at certain time points after administration of DVC1-0101 indicate its future potential for improving QOL in patients with CLI.

The strength of this study was its experimental design and its role as the first clinical study of the effects of gene therapy with DVC1-0101 on QOL in patients with CLI. However, the small sample size, single-arm, and dose-escalation study design limits the ability to generalize from the results, and further randomized, late-phase trials are needed to confirm the value of these preliminary results. Data concerning the effects of angiogenesis on QOL are currently limited, and the implications of the current study are ambivalent with regard to the roles of angiogenesis and angioplasty in relation to different staging classifications of PAD.

In conclusion, this study suggests the ability of gene therapy using DVC1-0101 to preserve or improve long-term QOL in patients with CLI. The increasing availability of gene therapy thus holds great promise for the future treatment of CLI.

## Methods

### Patients and study design

QOL data for CLI patients who were enrolled in a single-centre, phase I/IIa, open-label, four-dose-escalating trial of DVC1-0101 were used in this study^13^. The study was conducted in Japan from 2006 to 2010 and all the participants originated from the same geographic area. Twelve CLI patients were divided into four dose cohorts (n = 3 each) who received 5 × 10^7^, 2 × 10^8^, 1 × 10^9^, and 5 × 10^9^ cell infectious units/60 kg body weight, respectively, in a dose-escalating manner. They were observed for 6 months and followed-up for up to 12 months. DVC1-0101 was administered at 30 sites with varying ischemic conditions in one limb per patient. The Japanese Guidelines for Clinical Trials of Gene Therapy issued by the Ministry of Health, Labour and Welfare require that only ‘no-option’ patients may be enrolled in first-in-man gene therapy studies, and only CLI patients in whom standard vascular interventions were contraindicated were therefore eligible to participate. Other inclusion criteria were a diagnosis of arteriosclerosis obliterans or thromboangiitis obliterans, with no evidence of malignancies, unstable cardiovascular disease, immunodeficiencies, or haemodialysis. Nine patients had a history of smoking, but enrolment required them to stop smoking at least 1 month before screening^13^.

### Short Form-36 health survey and NBS

The Short Form-36 version 2 (SF-36) health survey was used to evaluate QOL, including measures of health improvement, treatment effectiveness, and comparisons of disease burden across populations^22^,^23^,^24^. SF-36 includes eight domains: physical functioning, role-physical, bodily pain, general health, vitality, social functioning, role-emotional, and mental health. These eight domains can be aggregated into three summary measures: physical component summary, mental component summary, and role/social component summary. The QOL scores were evaluated using a NBS method that delivered standardized scores such that the mean general population scores were set at 50 for every dimension. The NBS score was calculated using the 2007 Japanese population norms as representative. SF-36 has been shown to be reliable and has been validated worldwide, and its Japanese version has also been validated^22^,^23^,^24^. SF-36 was performed pre-administration, and at 28 days, and 3, 6, and 12 months after gene administration.

### Physiological parameters

ABI and TBI are indicators of the haemodynamics in the lower legs and toes. These indexes are calculated as the ratio between the highest blood pressure in the right or left brachial artery and the highest blood pressure in the posterior tibial or dorsalis pedis and toes^25^. An ABI <0.9 mmHg indicates leg ischemia. ABI and TBI were measured as a potential correlational variable using an automatic oscillometric system (VS-1500A; Fukuoka Denshi, Tokyo, Japan). Pulse-volume recording was measured using the same oscillometric system and rated using a 3-point grading scale: − = flat, + = slight pulsation, and ++ = normal pulsation. ACD and change in ACD as measures of walking function were assessed using a treadmill test. Eight patients were eligible for the flat-slope test at 2.4 km/h, which was terminated at 300 m to avoid the risk of cardiovascular complications. ABI and ACD were measured pre-administration as baseline and every month up to 12 months after gene therapy administration. Pain was rated using a 5-point Likert scale to measure the level of pain in the ischemic legs at rest: 1 = no pain, 2 = pain sometimes, 3 = occasionally need pain medication, 4 = always need pain medication, and 5 = cannot sleep at night even with pain medication. The laser Doppler perfusion index was measured using a laser Doppler perfusion index analyser (Moor Instruments, Denver, UK), and data only from the treated leg were used in the current study. Blinded dermatologists used thermography to measure the temperature of the skin of the feet, controlling for constant room temperature and allowing an interval after the removal of the patients’ socks and shoes. Differences in foot pad temperatures were calculated in degrees Celsius by subtracting the temperature of the untreated limb from the temperature of the treated limb.

### Ethics

Ethical approval for this clinical trial was granted by the Kyushu University Institutional Review Board for Clinical Trials, the Health Sciences Council of the Japan Ministry of Health, Labour and Welfare, and Biosafety Committees, according to national regulations and approved guidelines. Written informed consent was obtained from all subjects before data collection. This trial was registered with University Hospital Medical Information Network Center (Tokyo, Japan), UMIN-ID C000000404.

### Data analysis

SF-36 QOL scores were calculated using the Japanese SF-36 scoring program (v.3; QualityMetric Inc., Lincoln, RI, USA). Differences in baseline QOL scores in relation to characteristic variables were analysed by one-way analysis of variance (ANOVA). Differences between QOL scores before and after administration of DVC1-0101 and dose-related effects on QOL were analysed by Wilcoxon’s signed-rank tests. The influences of characteristic variables on the effects of DVC1-0101 on QOL were investigated by one-way ANOVA. Pearson’s correlation coefficient was used to examine the correlations between increases in QOL and ACD, ABI, and pain scale pre- and post-administration. Values of P < 0.05 were considered significant. All analyses were carried out using the Statistical Package for JMP software (v.11; SAS Institute Inc., Cary, NC, USA).

## Additional Information

**How to cite this article**: Matsumoto, T. *et al*. Improved quality of life in patients with no-option critical limb ischemia undergoing gene therapy with DVC1-0101. *Sci. Rep.* **6**, 30035; doi: 10.1038/srep30035 (2016).

## Supplementary Material

Supplementary Information

## Figures and Tables

**Table 1:** Baseline Short Form-36 norm-based scores in relation to characteristic variables (n = 12).

	ASO (n = 10)		TAO (n = 2)			Fontaine III (n = 9)		Fontaine IV (n = 3)		
	mean	SD	mean	SD	P value	mean	SD	mean	SD	P value
PF_N	5.86	12.06	36.19	5.11	0.0302	7.71	12.69	20.54	24.57	0.4012
RP_N	16.51	11.17	29.14	4.70	0.1258	16.58	11.84	24.71	8.36	0.3006
BP_N	33.23	6.91	33.14	16.42	0.9140	33.43	7.30	32.54	11.65	1.0000
GH_N	39.28	6.15	42.32	6.41	0.4465	38.15	5.95	44.72	2.82	0.0920
VT_N	36.34	11.80	46.62	22.71	0.4505	31.63	6.81	57.32	6.68	0.0122
SF_N	28.67	12.96	47.35	13.67	0.1514	29.82	11.51	37.69	23.23	0.5062
RE_N	31.09	11.27	49.83	8.83	0.0616	29.24	8.86	49.13	12.02	0.0295
MH_N	39.74	10.90	53.16	13.28	0.1814	37.81	9.56	54.51	9.68	0.0441
PCS	16.15	10.54	26.92	1.22	0.1974	18.03	8.54	17.70	17.48	0.6439
MCS	52.67	9.12	53.13	18.25	1.0000	49.24	8.30	63.29	6.58	0.0335
RCS	29.04	10.82	49.61	5.50	0.0317	29.11	12.26	42.54	9.52	0.1655

Abbreviations: ASO, arteriosclerosis obliterans; TAO, thromboangiitis obliterans; SD, standard deviation; PF_N, norm-based physical functioning; RP_N, norm-based role-physical; BP_N, norm-based bodily pain; GH_N, norm-based general health; VT_N, norm-based vitality; SF_N, norm-based social functioning; RE_N, norm-based role-emotional; MH_N, norm-based mental health; PCS, physical component summary; MCS, mental component summary; RCS, role/social component summary.

**Table 2:** Changes in Short Form-36 norm-based scores in critical limb ischemia patients receiving gene therapy (n = 10)^†^.

	Pre-treatment		1 month			3 months			6 month			12 months		
	mean (SD)		mean (SD)			mean (SD)			mean (SD)			mean (SD)		
PF_N	10.9	15.7	13.4	16.2		17.8	19.3	^**^	17.3	19.0	*	16.3	13.1	
RP_N	18.2	12.2	21.5	15.6		29.8	13.3	^**^	26.5	10.6	**	30.5	10.6	*
BP_N	34.6	7.8	37.2	9.9	^*^	41.3	9.0	^**^	42.0	8.1	**	42.3	9.8	*
GH_N	39.0	6.3	39.4	9.0		41.1	9.8		41.8	6.7		4.2	7.4	
VT_N	34.7	11.7	42.8	7.8	^*^	41.5	11.5		39.0	8.4	*	42.4	8.4	*
SF_N	32.5	13.8	31.2	13.9		37.7	18.0		39.0	13.8		35.8	11.8	
RE_N	31.9	11.9	29.4	13.6		31.9	14.3		33.4	10.2		31.5	12.8	
MH_N	40.3	11.9	40.0	7.0		41.4	10.8		40.3	8.5		39.7	9.2	
PCS	19.0	8.6	22.8	11.0		27.0	12.4		26.3	11.9	*	27.3	9.5	**
MCS	50.9	9.5	53.9	7.6		52.0	11.5		52.0	5.1		51.6	8.0	
RCS	31.6	13.9	28.6	13.7		34.8	13.9		34.7	12.0		33.9	13.6	

^*^P < 0.05. ^**^P < 0.01. ^†^As amputation strongly affects patient quality of life, data from the two patients who underwent amputation after administration of DVC1-0101 were excluded. Abbreviations: SD, standard deviation; PF_N, norm-based physical functioning; RP_N, norm-based role-physical; BP_N, norm-based bodily pain; GH_N, norm-based general health; VT_N, norm-based vitality; SF_N, norm-based social functioning; RE_N, norm-based role-emotional; MH_N, norm-based mental health; PCS, physical component summary; MCS, mental component summary; RCS, role/social component summary.

**Table 3:** Changes in Short Form-36 norm-based scores in relation to low- and high-dose DVC1-0101 (n = 12).

	1 month		3 months		6 months		12 months		
	Low	High	Low	High	Low	High	Low	High	P value
PF_N	0.50	−0.05	2.13	0.25	0.96	0.15	0.43	0.15	0.0294
RP_N	0.13	0.03	0.65	1.05	0.08	0.73	−0.03	1.08	0.1939
BP_N	0.10	0.08	0.03	0.19	0.25	0.26	0.25	0.32	0.4678
GH_N	−0.01	−0.05	−0.01	−0.02	0.12	0.03	0.06	0.01	0.3094
VT_N	0.27	0.04	−0.20	0.04	0.08	0.12	0.18	0.13	0.6631
SF_N	−0.03	−0.10	−0.03	0.14	0.03	0.21	−0.01	0.10	0.2425
RE_N	−0.02	−0.20	−0.04	0.02	−0.06	−0.01	−0.09	0.03	0.3123
MH_N	0.08	−0.12	0.00	−0.13	−0.01	−0.01	0.08	−0.09	0.0408

Abbreviations: PF_N, norm-based physical functioning; RP_N, norm-based role-physical; BP_N, norm-based bodily pain; GH_N, norm-based general health; VT_N, norm-based vitality; SF_N, norm-based social functioning; RE_N, norm-based role-emotional; MH_N, norm-based mental health.
