# Supplementary material for: Improved quality of life in patients with no-option critical limb ischemia undergoing gene therapy with DVC1-0101
Source: Sci Rep. 2016 Jul 15;6:30035. doi: 10.1038/srep30035 (PMC4945920; doi:10.1038/srep30035)
Supplement: Supplementary Information [file srep30035-s1.pdf]

Improved quality of life in patients with no-option critical limb ischemia undergoing gene therapy with DVC1-0101

Takuya Matsumoto[1], Michiko Tanaka[2], Keiji Yoshiya[1], Ryosuke Yoshiga[1], Yutaka Matsubara[1], Kumi Horiuchi-Yoshida[2], Yoshikazu Yonemitsu[2*] & Yoshihiko Maehara[1]

[1] Department of Surgery and Science, Graduate School of Medical Science, Kyushu University, 3-1-1 Maidashi, Higashi-ku, Fukuoka 812-8582, Japan

[2] R&D Laboratory for Innovative Biotherapeutics Science, Graduate School of Pharmaceutical Sciences, Kyushu University, Rm. 601, Collaborative Research Station I, 3-1-1 Maidashi, Higashi-ku, Fukuoka 812-8582, Japan

*Corresponding author

Supplementary Table S1 Baseline characteristics (n = 12)

Stage	Case number	Age	Sex	Smoking history	Clinical characteristics										Medical history		
					Diag-nosis	Ulcer	Fontaine/ Rutherford	Pain scale	ABI	TBI	PVR	ACD (m)	LDPI	Thermo-graphy	Revascula-rization	MI/ angina	stroke
1	102	59	M	+	ASO	−	III/II-4	2	0.07	0.07	−	67	43.4	−5	+	−	−
	103	74	M	+	ASO	+	IV/III-5	4	0.08	NA	−	61	22.6	−7	+	−	+
	105	66	M	−	ASO	+	IV/III-5	4	0.11	NA	−	NA	40.0	−4	+	−	−
2	201	65	F	−	ASO	−	III/II-4	2	0.61	0.10	−	74	114.0	0	−	−	−
	203	58	M	+	ASO	−	III/II-4	2	0.61	0.54	++	46	46.3	0	−	−	−
	204	76	F	−	ASO	−	III/II-4	2	0.38	0.05	−	NA	15.6	1.5	+	+	+
3	303	82	M	+	ASO	−	III/II-4	2	0.58	0.07	+	NA	17.2	−2	+	−	+
	304	58	M	+	ASO	−	III/II-4	3	0.38	0.10	−	122	30.4	−4.5	+	+	+
	305	83	M	+	ASO	−	III/II-4	2	0.54	0.08	−	96	20.3	1	+	+	+
4	401	48	M	+	TAO	−	III/II-4	2	0.70	0.06	+	243	60.4	−4.5	−	−	−
	403	54	M	+	TAO	+	IV/III-5	3	1.09	0.08	−	≧300	21.8	−8	+	+	+
	405	57	M	+	ASO	−	III/II-4	4	0.75	0.07	−	NA	39.5	−2	+	+	−

Abbreviations: ABI, ankle–brachial index; TBI, toe–brachial index; PVR, pulse-volume recording; ACD, absolute claudication distance; LDPI, laser Doppler perfusion index; MI, myocardial infarction; M, male; F, female; ASO, arteriosclerosis obliterans; TAO, thromboangiitis obliterans; NA, not applicable.

Supplementary Table S2 Relationships between baseline characteristics and changes in Short Form-36 summary values in critical limb ischemia patients receiving gene therapy (n = 12)

		Pre-treatment			1 month			3 months			6 months			12 months		
Diagnosis (ASO: TAO)	PCS	10.77	(−6.51–28.04)		12.03	(−9.59–33.65)		19.96	(−2.90–42.82)	*	6.99	(−12.06–26.04)		6.03	(−10.38–22.43)	
	MCS	.46	(−17.49–18.40)		−9.05	(−20.97–2.87)		−8.52	(−27.45–10.41)		0.24	(−11.97–12.45)		−1.13	(−20.21–17.96)	
	RCS	20.57	(2.60–38.54)	*	16.58	(−7.39–40.54)		15.94	(−4.02–35.90)		13.91	(−10.14–37.96)		6.55	(−20.41–33.50)	
Age (years) median = 65 (<65: ≥65)	PCS	2.27	(−11.9–16.45)		−3.41	(−20.80–13.98)		−16.48	(−33.12–0.16)	*	−3.02	(−17.74–11.70)		−5.46	(−17.68–6.76)	
	MCS	−3.25	(−16.62–10.12)		8.27	(−0.12–16.66)	*	12.17	(−0.15–24.49)	*	6.64	(−1.32–14.60)	*	9.49	(−3.31–22.29)	
	RCS	−8.66	(−25.01–7.68)		−6.95	(−26.50–12.60)		4.69	(−12.30–21.69)		−1.51	(−21.11–18.10)		−4.28	(−24.73–16.17)	
Sex (male vs. female)	PCS	7.98	(−10.03–25.99)		5.77	(−17.10–28.63)		4.24	(−22.44–30.91)		11.20	(−6.82–29.22)		2.39	(−14.47–19.26)	
	MCS	−13.6	(−28.76–1.62)		3.07	(−10.27–16.41)		15.62	(−0.90–32.15)		−2.79	(−14.85–9.27)		0.56	(−18.54–19.66)	
	RCS	8.27	(−14.07–30.60)		12.42	(−12.76–37.61)		10.18	(−11.57–31.93)	*	13.09	(−11.19–37.37)		7.07	(−19.83–33.96)	
Fontaine (III vs. IV)	PCS	−0.33	(−16.57–15.91)		−1.30	(−21.27–18.67)		−2.22	(−25.27–20.83)		5.68	(−10.77–22.14)		−8.44	(−21.76–4.88)	
	MCS	14.05	(−2.20–25.91)	*	3.37	(−8.01–14.76)		−1.13	(−18.20–15.94)		10.52	(3.07–17.98)	*	16.33	(4.59–28.08)	*
	RCS	13.43	(−4.04–30.90)		8.05	(−14.19–30.28)		10.41	(−7.89–28.71)		−7.83	(−29.49–13.84)		−13.94	(−35.33–7.45)	
Medical history of MI (+ vs. −)	PCS	−3.08	(−17.18–11.02)		−0.56	(−18.11–16.99)		−6.59	(−26.34–13.16)		−2.64	(−17.39–12.12)		−4.24	(−16.70–8.22)	
	MCS	3.19	(−10.19–16.57)		5.04	(−4.54–14.62)		9.87	(−3.43–23.17)		4.24	(−4.50–12.97)		10.19	(−2.34–22.72)	
	RCS	4.54	(−12.61–21.70)		4.33	(−15.59–24.25)		−2.05	(−19.30–15.20)		2.63	(−16.91–22.18)		9.85	(−9.63–29.32)	
Dose (high vs. low)	PCS	−2.30	(11.68–16.27)		−2.31	(14.92–19.55)		−5.92	(13.65–25.49)		3.90	(18.31–10.50)		−5.01	(7.12–17.14)	
	MCS	−0.04	(−13.42–13.34)		6.20	(−2.88–15.28)		12.45	(0.53–24.37)	*	1.80	(−7.22–10.81)		8.74	(−4.10–21.58)	
	RCS	6.68	(−9.86–23.23)		10.17	(−8.37–28.70)		−3.71	(−20.58–13.16)		−3.42	(−22.63–15.79)		−5.13	(−25.19–14.93)	

* P <.05. Values are given as sum of mean (95% confidence interval). Abbreviations: ASO, arteriosclerosis obliterans; TAO, thromboangiitis obliterans; MI, myocardial infarction; PCS, physical component summary; MCS, mental component summary; RCS, role/social component summary.

Supplementary Table S3 Correlations between change rate in improvement of Short Form-36 summary scores and improvement of change rate in objective findings

		1 month		3 months			6 months			12 months		
		r	*p*	*r*	*p*		*r*	*p*		*r*	*p*	
ACD	PCS	−.30	.624	.20	.747		.80	.104		−.10	.873	
(n = 5) †	MCS	.70	.188	.20	.747		−.20	.747		.00	>.95	
	RCS	.50	.391	.50	.391		−.50	.391		.30	.624	
ABI	PCS	−.35	.328	.43	.215		.01	.987		.10	.777	
(n = 10) ‡	MCS	.44	.200	−.16	.652		.07	.855		.21	.556	
	RCS	.43	.215	−.42	.229		−.09	.803		−.09	.803	
TBI	PCS	−.01	.973	.09	.800		−.26	.466		.88	.001	*
(n = 10) ‡	MCS	.10	.776	.18	.623		.35	.318		−.69	.028	*
	RCS	.43	.215	−.42	.229		−.09	.803		.14	.700	
pain scale	PCS	.60	.069	.32	.371		.16	.656				
(n = 10) ‡	MCS	−.58	.082	−.64	.048	*	−.21	.554				
	RCS	−.45	.197	−.40	.249		−.65	.041	*			
thermography	PCS	.04	.906	.61	.081		.86	.001	*			
(n = 10) ‡	MCS	.10	.774	−.32	.398		−.71	.023	*			
	RCS	.01	.987	−.46	.215		−.54	.106				

* P <.05. † Six patients who did not have thromboangiitis obliterans were eligible for the treadmill test. One patient was excluded because of a lack of pre-treatment data as they had very severe rest pain. ‡ As amputation strongly affects patient quality of life, data from the two patients who underwent amputation were excluded. Abbreviations: ACD, absolute claudication distance; ABI, ankle–brachial index; TBI, toe–brachial index; PCS, physical component summary; MCS, mental component summary; RCS, role/social component summary.
